# Effects of Statistical and Narrative Health Claims on Consumer Food Product Evaluation

**DOI:** 10.3389/fpsyg.2020.541716

**Published:** 2021-01-11

**Authors:** Hung-Chou Lin, Sheng-Hsien Lee

**Affiliations:** ^1^Department of Adult and Continuing Education, National Taiwan Normal University, Taipei, Taiwan; ^2^General Education Center, National Defense University, Taoyuan City, Taiwan

**Keywords:** health claims, health knowledge, statistical messages, need for cognition, narrative messages

## Abstract

This research aims at exploring the underlying mechanisms how consumers respond to statistical and narrative health claims when they evaluate food products. Moreover, personality traits and product-related information are also incorporated to discuss their effects on the relationship between message types and consumers’ food product evaluation. The results indicate that statistical health claims are more persuasive than narrative health claims. In addition, the results show that individuals’ health knowledge, NFC moderate the relationship between message types and product evaluation. It argues that individuals with limited health knowledge evaluate food product more favorably when statistical health claims are used, while individuals with more health knowledge evaluate food product more favorably when narrative health claims are used. Moreover, it reveals that individuals with high NFC evaluate food product more favorably when statistical health claims are used, while individuals with low NFC evaluate food product more favorably when narrative health claims are used.

## Introduction

Consumers are increasingly wary of the health effects of the food they eat. Indeed, a recent study finds that labeling and product information are important factors in consumers’ decisions regarding whether to try the products (Li et al., 2019). In response, the food industry now employs more thorough nutrition labeling, and more frequent claims tout the health benefits of foods. Health claims can help consumers understand how food products affect their health and thus maintain healthy eating habits, thereby improving general public health (Leathwood et al., 2007). Studies (Balasubramanian and Cole, 2002; Kozup et al., 2003; Bialkova and van Trijp, 2011; Miller and Cassady, 2012; Tanner et al., 2019) have considered the effects of health claims on motivation, health beliefs, on purchase intention. Roe et al. (1999) discovered that consumers viewed products as being healthier if the products presented nutritional or health-related claims. Moreover, such claims may be particularly effective when they are the only health-related information on the packaging (Kozup et al., 2003; Van Trijp and Van der Lans, 2007).

Consumer interest in health claims depends on several factors, such as the format and content of messages (Mazis and Raymond, 1997). When presenting health information, multiple messages or forms of evidence can be used. Both statistical and narrative messages have attracted considerable attention. Statistical messages include “quantified descriptions of events, persons, places, or other phenomena” (Church and Wilbanks, 1986, p. 108). The persuasiveness of statistics potentially depends on the sample size because larger samples suggest greater objectivity. Narrative messages typically present information in a personal way (Vanderford et al., 1992; Ellinson and Buzzanell, 1999; Arneson and Query, 2001), and narrative messages are defined as “case stories or examples to indicate that the conclusion offered by the communicator is true” (Allen and Preiss, 1997). The difference in the characteristics of narrative and statistical presentation raises the question of which message type is more persuasive. Much research has examined the comparative effects of these two types of message. Kahneman and Tversky (1973), Kazoleas (1993), and Taylor and Thompson (1982) have declared that statistical messages are less persuasive regarding judgments and attitudes; by contrast, some authors indicate that statistical messages provide more accurate product information (Dickson, 1982) and thus are more influential on consumer beliefs (Baesler and Burgoon, 1994). Whether statistical or narrative health claims can enhance the persuasiveness of communication remains unclear. Therefore, the present study explored whether statistical or narrative messages enhance the persuasiveness of health claims regarding food products. Moreover, Skurka et al. (2020) compare the concepts of individual and collective narrative messages and find that subjects’ “reactions to the collective (vs. individual) versions could have been more intuitive and outside conscious processing.” In other words, the collective narrative messages produce less external thinking and more automatic reactions. Hence, collective narrative messages will be utilized in the present research for developing experimental materials.

This study therefore investigated the individual differences between statistical and narrative health claims. In particular, individual health knowledge and need for cognition (NFC) were incorporated to examine their effects on consumer evaluations of food products. Individual knowledge is crucial in purchasing decisions. The knowledge individuals possess affects their information search behavior (Brucks, 1985) as well as their processing of that information, subsequent decision-making, and ultimate purchasing intention (Lin and Chen, 2006). Individuals with different amounts of health knowledge are likely to react differently to messages and information, but how they respond differently to statistical and narrative health claims when evaluating food products is unknown. Individuals’ NFC influences their information processing. Individuals with high NFC have an increased motivation to seek, collect, and analyze information; by contrast, people with lower NFC expend fewer cognitive resources on these tasks and more prone to employ heuristics to process messages. Because individuals with different NFC levels process information differently, their responses to statistical and narrative health claims during food product evaluation are worthy of investigation.

## Literature Review

### Statistical and Narrative Health Claims and Consumer Food Product Evaluation

Statistical and narrative are the dominant message types used to present health claims about food products. Statistical messages summarize numerous cases and typically present summary statistics for a population, whereas narrative messages are conveyed in a personal format (Arneson and Query, 2001). Because narrative and statistical messages have different characteristics, they may elicit disparate responses. For instance, narrative messages result in affective reactions (Kopfman et al., 1998), whereas statistical messages provoke more cognitive responses. Researchers have compared the effects of statistical and narrative messages, but whether statistical or narrative health claims are more persuasive remains unclear. Kazoleas (1993) and Taylor and Thompson (1982) have revealed that statistical messages are less persuasive than are narrative messages; by contrast, Allen and Preiss (1997) demonstrated that statistical messages are slightly more effective than are narrative messages. Baesler and Burgoon (1994) argued that compared with no message, both message types are initially persuasive, but in the long term, statistical messages are more persuasive. Zebregs et al. (2015) showed that whereas statistical messages have more impact on beliefs and attitudes, narrative messages lead to higher levels of intention. Although somewhat inconclusive, previous findings have indicated a relatively complex relationship between message type and communication effects in marketing. Hence, the present research explored whether and in which conditions statistical or narrative health claims can increase the persuasiveness of communication and enhance consumers’ evaluations of food products.

The elaboration likelihood model (ELM) of persuasion presented by Petty and Cacioppo (1986) can be a useful framework for evaluating the persuasiveness of statistical and narrative messages. Attitude change occurs *via* central and peripheral routes. When forming attitudes about a product or advertisement, individuals favoring the central route critically consider issue-related arguments and their merits and relevance, whereas the peripheral route employs less cognitive activity and favors heuristics, such as the physical attractiveness of people endorsing the product or the number of claims made. Ability to elaborate and individual motivation affect the elaboration likelihood; for instance, motivated individuals can process information carefully. Such individuals are influenced primarily by the characteristics of messages, about which their thinking elaborates. When individuals lack the motivation or capability required to think this way, they process information superficially and are influenced mostly by peripheral cues, that is, those regarding the origin, rather than the content, of the information. Individuals increasingly believe that their health is directly influenced by their food (Young, 2000; Mollet and Rowland, 2002; Xhakollari et al., 2019), and such individuals are willing to modify their eating habits in pursuit of a healthier lifestyle (Niva, 2007). Therefore, when evaluating health claims about food products, these individuals are motivated to process information relevant to their health. Hence, individuals tend to critically consider issue-related arguments. In this case, statistical health claims are more persuasive than narrative health claims. That is, individuals evaluate statistical health claims more favorably than narrative health claims. Therefore, hypothesis 1 (H1) is proposed.

*H1*: Individuals evaluate food products with statistical health claims more favorably than those with narrative health claims.

### Effect of Health Knowledge on Statistical and Narrative Health Claims

Some studies have found that compared with those with less health knowledge, individuals with greater health knowledge are more likely to take advantage of health information and incorporate it into decision-making. Moorman and Matulich (1993) argued that health knowledge, alongside health motivation, promotes healthy behavioral responses. When evaluating food products, individuals tend to use health information in different ways depending on their level of health knowledge. For instance, greater health knowledge increases the likelihood of reading nutrition labels to make food purchase decisions (Kim et al., 2001). In addition, consumers are more likely to consider nutrition labels when the information they contain is simpler to comprehend (e.g., Moorman, 1996; Kemp et al., 2007). In general, knowledge facilitates information search, and more knowledgeable consumers are more likely to seek, obtain, and utilize relevant information.

Thunholm (2005) found that individuals with greater knowledge are more likely to make decisions quickly; moreover, they occasionally neglect useful information (Radecki and Jaccard, 1995) or cease the pursuit of new information (Wood and Lynch, 2002; Bhargave et al., 2016). However, individuals with little preexisting knowledge tend to make critical assessments and compare available options (Mitchell and Dacin, 1996).

The present study investigated how individuals with different levels of health knowledge respond to statistical and narrative health claims. We hypothesized that among individuals with limited health knowledge, the persuasive effect of statistical health claims is higher than that of narrative health claims. According to the ELM of persuasion and health knowledge literature, individuals with limited health knowledge are prone to the central route and view statistical health claims favorably because they are critically motivated to consider health claims and compare alternatives. By contrast, individuals with more health knowledge tend to overlook information and make quick decisions, leading them to take the peripheral route and view narrative health claims more favorably.

*H2*: Individual health knowledge and message type interact when individuals evaluate food product. Specifically, individuals with limited health knowledge evaluate statistical claims more favorably, whereas individuals with greater health knowledge evaluate narrative claims more favorably.

### Effect of Need for Cognition on Statistical and Narrative Health Claims

NFC is the tendency to engage in and enjoy cognitive activity (Cacioppo and Petty, 1982). Compared with individuals with low levels of NFC, individuals with high NFC more actively seek, obtain, and analyze information and utilize more cognitive resources for processing messages. Moreover, high-NFC individuals are less likely to employ heuristics to process messages (Peer and Gamliel, 2012) but more influenced by the value of the evidence and argumentation of the message compared with low-NFC individuals. By contrast, low-NFC individuals avoid strenuous cognitive activity and utilize fewer cognitive resources to interpret messages; thus, they are likely to process messages heuristically. Moreover, they have low cognitive motivation and stray away from situations requiring substantial elaboration; high-NFC individuals, by contrast, are attracted to such situations (Cacioppo et al., 1984).

The present study investigated how individuals with different NFC levels respond to statistical and narrative health claims. Statistical health claims were predicted to have a greater persuasive effect than narrative health claims have on individuals with high NFC. Statistical health claims are more informative than are narrative health claims; they also attract attention more effectively and elicit a deeper level of information processing. Because high-NFC individuals are more inclined to think critically, they evaluate statistical health messages more favorably than they evaluate narrative ones. Moreover, low-NFC individuals are more prone to use heuristics and more influenced by surface cues than are those with high NFC. Narrative health claims may be appealing and persuasive to individuals with low levels of NFC because such messages do not require extensive cognitive processing. Individuals with low NFC should therefore perceive narrative health claims more favorably than statistical health claims. Hence, such individuals are expected to embrace narrative health claims, whereas individuals with high NFC should be more prone to influence by statistical claims.

*H3*: NFC and message type interact when individuals evaluate food products: individuals with high NFC evaluate food products more favorably when statistical health claims are used, whereas individuals with low NFC evaluate food products more favorably when narrative health claims are used.

## Study 1

Study 1 tested H1—whether individuals respond more favorably to statistical health claims than to narrative health claims. The participants in all three studies are all independent, as this study was a between-subject design.

### Method

**Participants, Experimental Design, and Procedure.** In total, 80 university students were recruited and randomly assigned to two experimental groups that received statistical or narrative health messages. Participants were then presented with an image of a food product. All participants were shown the same product with the same brand name. We used a fictitious brand name, Sunplus, to avoid preexisting brand preference or prejudice. Next, participants were asked to simply evaluate the product.

**Message Type Manipulation.** Either a statistical or narrative health claim was clearly stated. In order to capture more intuitive and more automatic reactions from participants, this study utilized collective narrative health claim for developing experimental materials. The statistical health claim stated that 95 of 100 customers are satisfied with Sunplus and that using a bottle a day improves digestive and immune function. By contrast, the narrative claim was that most customers recommend Sunplus after use and that using a bottle a day improves digestive and immune function.

**Dependent Measures.** The dependent measures used in Study 1 were the same as used for Studies 2 and 3. The dependent variables of this study include four items whereby they capture the major attitudinal components: affect, behavioral, and cognitive (Lutz, 1981) and are consistent with past literatures (Kozup et al., 2003). This study used a 7-point Likert scale to measure the participants’ preference. A 7-point scale with the endpoints labeled as absolutely not and absolutely assessed three items: attractive, convincing, and credible. Another item assessed participants’ intention to buy the product: “Can you imagine yourself buying this product?” Similarly, a 7-point scale with the endpoints labeled as absolutely not and absolutely assessed the participants’ intention.

### Results

One-way analysis of variance of food product evaluations revealed that products bearing statistical health claims were rated higher (*M* = 3.97) than those supported by narrative health claims (*M* = 3.30); thus, message type significantly affected food product evaluation [*F*(1, 78) = 7.847, *p* = 0.006]. On the other hand, a Levene’s test was conducted and shows the homogeneity of variance across treatment (*p* = 0.432). Study 1’s findings thus support H1.

## Study 2

Study 2 tested H2—whether individuals with less health knowledge more favorably evaluate food products bearing statistical health claims and whether individuals with more health knowledge receive narrative claims more favorably.

### Method

**Participants, Experimental Design, and Procedure.** In total, 121 university students were recruited, and a 2 (message types: statistical vs. narrative) × 2 (health knowledge: high vs. low) experimental design was used. Past literature shows that each treatment should obtain a minimal of 30 responses to ensure the stability of statistical analysis (Cheung and Slavin, 2013). Participants completed the dietary and health knowledge subscale of the Diet and Health Knowledge Survey (DHKS). We adopted a median split to divide the participants into high and low health knowledge, which is a common method in experimental study (Dar et al., 2019; Mekari et al., 2019). On the basis of the health knowledge scale ratings, participants were then randomly distributed to the experimental conditions of statistical or narrative messages. The design was a two-factor, two-level, between-subject design. Study 2 employed the same message type manipulation and product appraisal used in Study 1.

**Health Knowledge and Dependent Measures.** The dietary and health knowledge subscale of the DHKS consists of 11 self-report items prompted by the same question asking whether each of the following actions is *very important* (4 points), *somewhat important* (3 points), *not too important* (2 points), or *not at all important* (1 point): (a) sodium or salt use only in moderation; (b) a diet low in saturated fat; (c) plenty of fruits and vegetables; (d) sugar consumption only in moderation; (e) adequate fiber; (f) food variety; (g) healthy weight; (h) a low-fat diet; (i) a low-cholesterol diet; (j) plenty of breads, cereals, rice, and pasta; and (k) two or more daily servings of dairy. The dependent measurements were identical to Study 1.

### Results

**FIGURE 1:**
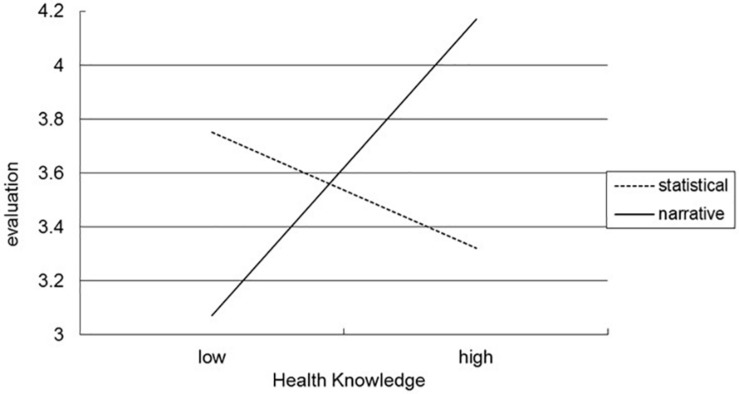
The moderating effect of health knowledge on message types and food product evaluation.

The results of two-way analysis of variance of food product evaluation revealed a two-way interaction between message type and health knowledge [*F*(1, 117) = 7.119, *p* = 0.009]; however, the main effects of message type [*F*(1, 117) = 0.007, *p* = 0.934] and health knowledge [*F*(1, 117) = 3.041, *p* = 0.084] were nonsignificant. As illustrated in Figure 1, participants with low health knowledge rated food products supported by statistical health claims (*M* = 3.75) higher than those by narrative health claims [*M* = 3.07; *F*(1, 59) = 4.532, *p* = 0.03705], whereas participants with high health knowledge rated food products with narrative health claims (*M* = 4.17) higher than they did those with statistic health claims [*M* = 3.32; *F*(1, 58) = 6.489, *p* = 0.014]. On the other hand, a Levene’s test was conducted and shows the homogeneity of variance across treatment (*p* = 0.542). Study 2’s findings thus support H2.

## Study 3

Study 3 tested H3—whether individuals with high NFC evaluate food products with statistical health claims more favorably, and individuals with low NFC evaluate food products with narrative health claims more favorably.

### Method

**Participants, Experimental Design, and Procedure.** In total, 129 university students were recruited, and a 2 (message types: statistical vs. narrative) × 2 (NFC: low vs. high) experimental design was used. Participants completed the NFC scale developed by Cacioppo et al. (1984); on the basis of these results, participants were randomly assigned to the experimental conditions of statistical or narrative messages. Study 3 followed the same message type manipulation and product evaluation procedures as the other two studies.

**NFC Measures.** To assess NFC, the short form of the Cacioppo et al. (1984) scale was employed. This scale tests both internal and test–retest reliability. The scale also demonstrated validity by predicting the degree of message-relevant thought in persuasion contexts (e.g., Cacioppo et al., 1986); moreover, it is reliable (alphas of approximately 0.90) and unidimensional (Cacioppo et al., 1986).

### Results

**FIGURE 2:**
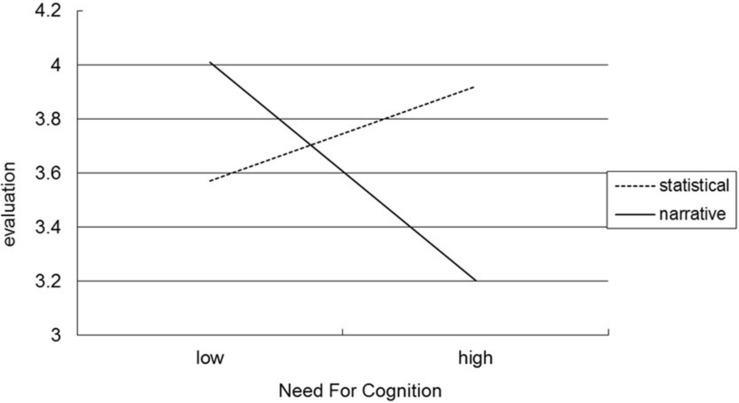
The moderating effect of need for cognition (NFC) on message types and food product evaluation.

The results of two-way analysis of variance of food product evaluation revealed a two-way interaction between message type and NFC [*F*(1, 125) = 8.238, *p* = 0.005]; however, the main effects of message type [*F*(1, 125) = 0.829, *p* = 0.364] and NFC [*F*(1, 125) = 1.303, *p* = 0.256] were nonsignificant. As illustrated in Figure 2, participants with low NFC rated food products with narrative health claims (*M* = 4.01) higher than those with statistical health claims [*M* = 3.57; *F*(1, 62) = 4.0326, *p* = 0.049], whereas participants with high NFC rated food products with statistical health claims (*M* = 3.93) higher than those with narrative health claims [*M* = 3.20; *F*(1, 63) = 6.327, *p* = 0.014]. Study 3’s findings thus support H3.

## Discussion and Implications

This study has both academic and practical implications. Although previous empirical consumer behavior research has investigated the effects of message type (e.g., Kahneman and Tversky, 1973; Nisbett and Borgida, 1975; Nisbett and Ross, 1980; Taylor and Thompson, 1982; Kazoleas, 1993; Kopfman et al., 1998), whether statistical or narrative is more persuasive remains unclear. Moreover, most studies on message types have focused on the effects of message types in advertising on consumers’ behavior, but how consumers respond to different message types on the package of a food product remains unknown. Therefore, the present study furthers the understanding of the effects of message types.

This study found that the ELM of persuasion accounts for the relationship between message type and consumer food product appraisals. The ELM of persuasion includes both the central and peripheral routes. Results of the present research indicate that individuals are motivated to process health-related information when they evaluate the health claims of food products. Within the framework of the ELM of persuasion, individuals thus tend to take the central route and consider issue-related arguments critically. Therefore, statistical health claims are more persuasive than narrative health claims. In other words, individuals evaluate statistical health claims more favorably than narrative health claims.

This study also found health knowledge and NFC moderate the effect of message type on food product evaluation. Individuals with limited health knowledge assess food products with statistical health claims more favorably, whereas individuals with more health knowledge judge food products with narrative health claims more favorably. Furthermore, individuals with high NFC judge food products with statistical health claims more favorably, whereas individuals with low NFC assess food products with narrative health claims more favorably. Such results have not been reported.

Regarding practical benefits, food product companies typically present favorable health claims on their products. However, the persuasiveness of statistical and narrative messages remains unclear. The following results of this study may benefit marketing practitioners.

First, statistical health claims appear more attractive than narrative health claims, generally. This observation indicates that individuals tend to consider issue-related arguments critically.

Second, our results indicate that the effect of message types is moderated by individual differences, implying that marketers should employ market segmentation together with careful selection of message type. Individuals with low health knowledge employ peripheral cues as evaluative reference points; thus, food products appear more attractive when a statistical health claim is used. By contrast, individuals with high health knowledge tend to employ rational thinking, such that they evaluate food products favorably when a narrative health claim is used.

Regarding future research, the present research combined attitudes and intentions into one dependent measure. However, the relationship between the two seemingly different concepts is worth further exploration in the future study. A proper construct development for intention as a dependent variable is also relevant in the future research of interest.

This study adopted controlled lab experiments in order to prevent effects from outside the research design, which will of course sacrifice the potential influence from situational factors, such as brands, endorsement, and promotions. To make the findings more applicable to the real world, future studies are advised to develop field experiments to include the brand effects and test their interactions in the ELM domain.

## Data Availability Statement

The original contributions presented in the study are included in the article/supplementary material, further inquiries can be directed to the corresponding author/s.

## Ethics Statement

Ethical review and approval was not required for the study on human participants in accordance with the local legislation and institutional requirements. Written informed consent from the participants was not required to participate in this study in accordance with the national legislation and the institutional requirements.

## Author Contributions

H-CL designed the study and drafted the manuscript. H-CL and S-HL collected and analyzed the data. Both authors contributed to the article and approved the submitted version.

## Conflict of Interest

The authors declare that the research was conducted in the absence of any commercial or financial relationships that could be construed as a potential conflict of interest.
